# ABCC1 protects skin dendritic cells from FITC-induced toxicity by efflux and extracellular glutathione buffering

**DOI:** 10.1073/pnas.2538155123

**Published:** 2026-03-05

**Authors:** Konrad Knöpper, Anshul Rao, Jinping An, Jason G. Cyster

**Affiliations:** ^a^HHMI and Department of Microbiology and Immunology, University of California, San Francisco, CA 94143

**Keywords:** dendritic cells, apoptosis, cell trafficking, skin, transporter

## Abstract

Dendritic cell (DC) migration is critical for initiating adaptive immune responses. Previous work suggested a role for ATP-binding cassette transporter C1 (ABCC1) in skin DC migration following cutaneous fluorescein isothiocyanate (FITC) exposure, but the precise mechanism involved was unclear. Here, we establish that the primary contribution of ABCC1 to skin DC function following FITC exposure is not modulation of migration, but enhancement of survival. Our findings demonstrate that ABCC1 operates on a dual level: Intracellularly, by transporting toxic FITC and fluorescein out of DCs, and extracellularly, by contributing to a glutathione (GSH) buffer zone that protects surrounding cells. DCs are particularly susceptible to FITC-mediated toxicity, possibly due to their high endocytic activity. This study elucidates the critical dependence of DCs on ABCC1 and extracellular GSH for resistance to toxic organic molecules and thereby identifies potential therapeutic avenues targeting ABCC1 to modulate immune responses.

## Significance

Adaptive immunity is critically dependent on dendritic cells (DCs) and their ability to take up foreign molecules for processing and presentation to T cells. DCs in the skin are exposed to a diversity of environmental chemicals, and whether they have mechanisms to protect themselves from chemical-induced toxicity has been unclear. In this work, we investigate the role of the multidrug resistance transporter ABCC1 in DC biology. We reveal that ABCC1 shields DCs from chemical poisoning following skin exposure to fluorescein isothiocyanate. ABCC1 is needed both cell intrinsically and cell extrinsically to achieve the full protective effect. This finding underscores a previously unrecognized reliance of DCs on ABCC1 function and opens new opportunities for therapeutic manipulation of these cells.

DCs in the skin continually sample their microenvironment for antigens and for the presence of pathogen-associated molecular patterns (PAMPs), damage-associated molecular patterns (DAMPs), and inflammatory cytokines. Upon exposure to such stimuli, they upregulate CCR7 and costimulatory molecules, migrate into skin lymphatics in a CCR7-dependent manner and subsequently into the T zone of the draining lymph node (LN) (1). Here, they present antigen-derived peptides on MHC molecules to prime T cell responses. The barrier location of DCs together with their endocytic activity and their need for metabolically intense behaviors may make them particularly sensitive to environmental chemicals.

ATP-binding cassette transporter C1 (ABCC1, also known as multidrug resistance transporter protein-1 or MRP1) has a well-defined role in effluxing a range of anionic molecules out of cells, including various drugs and environmental chemicals, often as glutathione (GSH) conjugates (2). ABCC1 also effluxes the oxidized form of GSH, GSSG, and this helps reduce potentially toxic intracellular buildup of this metabolite. Additionally, ABCC1 contributes to the export of GSH into the extracellular milieu (2). Another important substrate of ABCC1 is the GSH conjugated bioactive lipid leukotriene C4 (LTC4) (2). In recent work, we established ABCC1 is a transporter of S-geranylgeranyl-L-glutathione (GGG), a ligand for P2RY8, in lymphoid tissues (3). Whether ABCC1 has a role in exporting environmental chemicals from DCs and thereby protecting their immune function is unknown.

The fluorescein-coupled form of isothiocyanate (FITC) has been used for over four decades to study skin DC activation and migration to skin-draining LNs (sdLNs) (4, 5). Isothiocyanates (ITCs) are chemically reactive and can couple to thiol and amino groups (6, 7). Following topical application, various types of skin-derived DCs that harbor FITC-labeled proteins begin appearing in the sdLN within several hours and continue to accumulate over 2 to 3 d (8). The mechanism by which FITC induces DC maturation is not well understood but given its chemical reactivity it may involve triggering of DAMP-sensing pathways.

Here, we pursue the basis for the finding that ABCC1 is required for skin DC migration to LNs following exposure to FITC (9–11). ABCC1 is highly expressed by DCs, and we found that the transporter protects DCs from toxicity and cell death induced by FITC and fluorescein. DCs were more sensitive than other CD45+ cell types to this toxicity and more dependent on ABCC1 for survival. Bone marrow (BM) chimera studies provided evidence that ABCC1 contributes both intrinsically and extrinsically to DC resistance to FITC-induced toxicity. These findings indicate that DCs have a high dependence on ABCC1-mediated molecular efflux and ABCC1-dependent extracellular GSH to sustain their viability and function following tissue exposure to some classes of reactive chemicals.

## Results

**Fig. 1.**
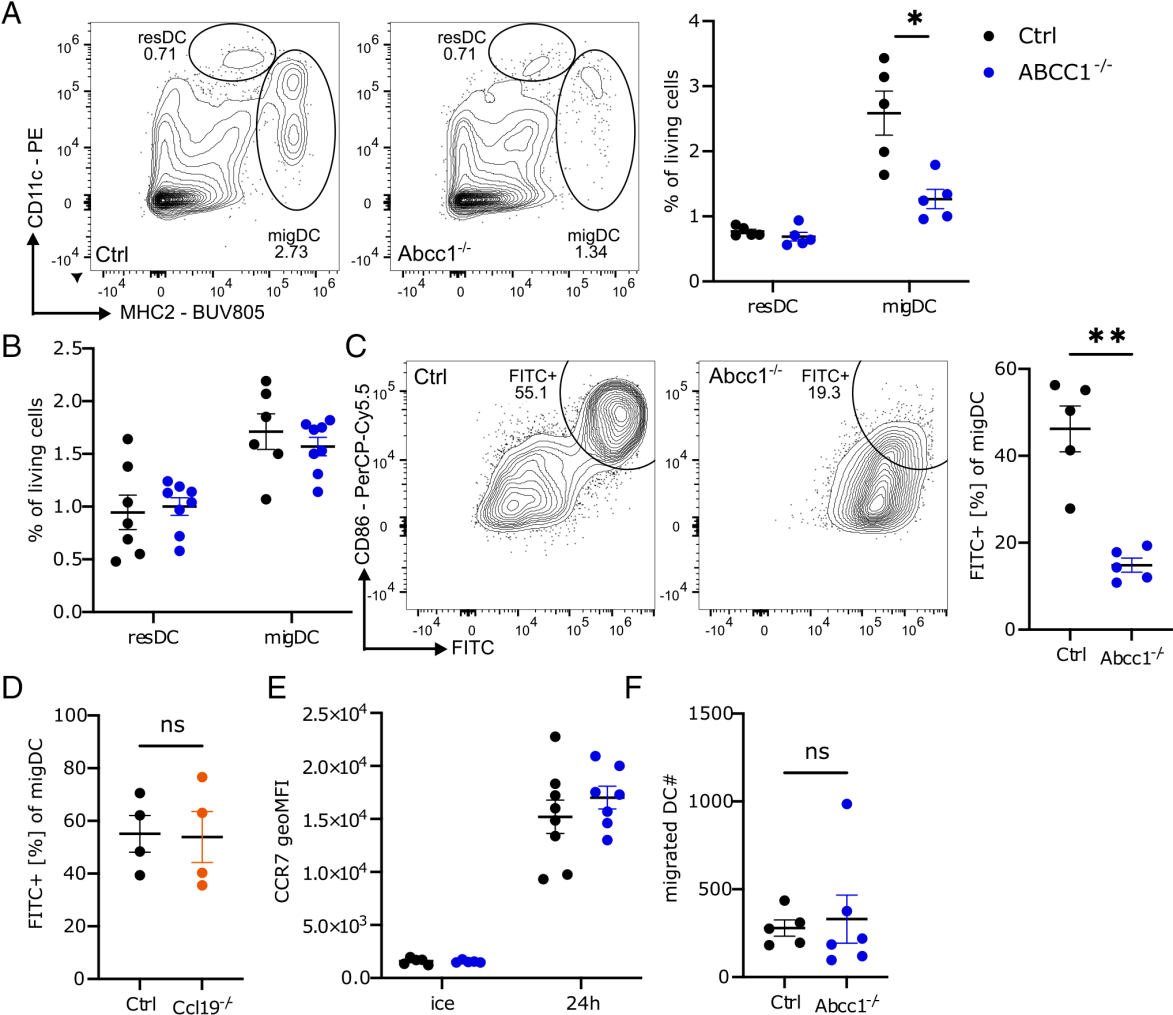
ABCC1 promotes accumulation of migratory DCs in the sdLN following FITC treatment in a CCL19 and migration independent manner. (*A*) Representative flow cytometry plot and quantification of sdLN DC subsets in control and ABCC1-deficient mice. sdLN were analyzed 18 h after FITC treatment on the skin. (*B*) Quantification of sdLN resident and migratory DC subsets in control and ABCC1-deficient naive mice. (*C*) Representative flow cytometry plot and quantification of sdLN FITC+ migratory DCs in control and ABCC1-deficient mice. sdLN were analyzed 18 h after FITC treatment on the skin. (*D*) Quantification of sdLN FITC+ migratory DCs in control and CCL19-deficient mice. sdLN were analyzed 18 h after FITC treatment on the skin. (*E*) Quantification of CCR7 geoMFI staining from spleen DCs. DCs were isolated and either matured for 24 h in culture media or kept on ice. (*F*) Quantification of migrated DCs from ear-crawl-out assay after 24 h. All data are representative of at least two independent experiments (n ≧ 3 mice per experiment). Error bars indicate SEM, and statistical analysis was done with Student’s *t* test. ns = not significant; *P-value < 0.05; **P-value < 0.01.

Prior work found that migratory DC accumulation in sdLNs 18 h following FITC application on the skin was reduced in ABCC1-deficient mice (9). Using a similar FITC treatment approach we confirmed this observation in a distinct line of ABCC1-deficient mice (Fig. 1*A* and *SI Appendix*, Fig. S1*A*). This reduction in migratory DCs was not evident at homeostasis, and there was also no change in resident DCs or Langerhans cells (LCs) (Fig. 1*B* and *SI Appendix*, Fig. S1 *B*–*E*). The reduction in sdLN migratory DCs after FITC application was predominantly attributable to a diminished appearance of FITC+ migratory DCs (Fig. 1*C* and *SI Appendix*, Fig. S1*F*). At this 18-h time point, the migratory skin DCs are conventional DCs as LCs have a delayed migratory behavior and take several days to reach the sdLN (12, 13). While a defect in ABCC1-deficient skin DC migration was previously suggested to involve impaired responsiveness to the CCR7 ligand CCL19 (9), other work found that CCL19 was dispensable for DC migration following FITC-mediated activation (14). Consistent with the latter findings, we did not observe a significant difference in the appearance of FITC+ DCs in the sdLN when comparing CCL19-deficient and control mice (Fig. 1*D*). Thus, the ABCC1-dependent DC phenotype appeared unlikely to be due to altered CCL19-directed migration.

In accordance with this observation, we detected comparable surface CCR7 expression on ex vivo matured spleen DCs from ABCC1-deficient and control mice (Fig. 1*E*). Furthermore, analysis of DC migration via crawl-out assay from ear skin explants revealed no significant differences between ABCC1-deficient and control mouse skin (Fig. 1*F*). These observations collectively led us to consider that the migration of DCs may not be directly compromised by ABCC1 deficiency and that the reduction in FITC+ DCs in sdLN reflected other roles for the transporter.

**Fig. 2.**
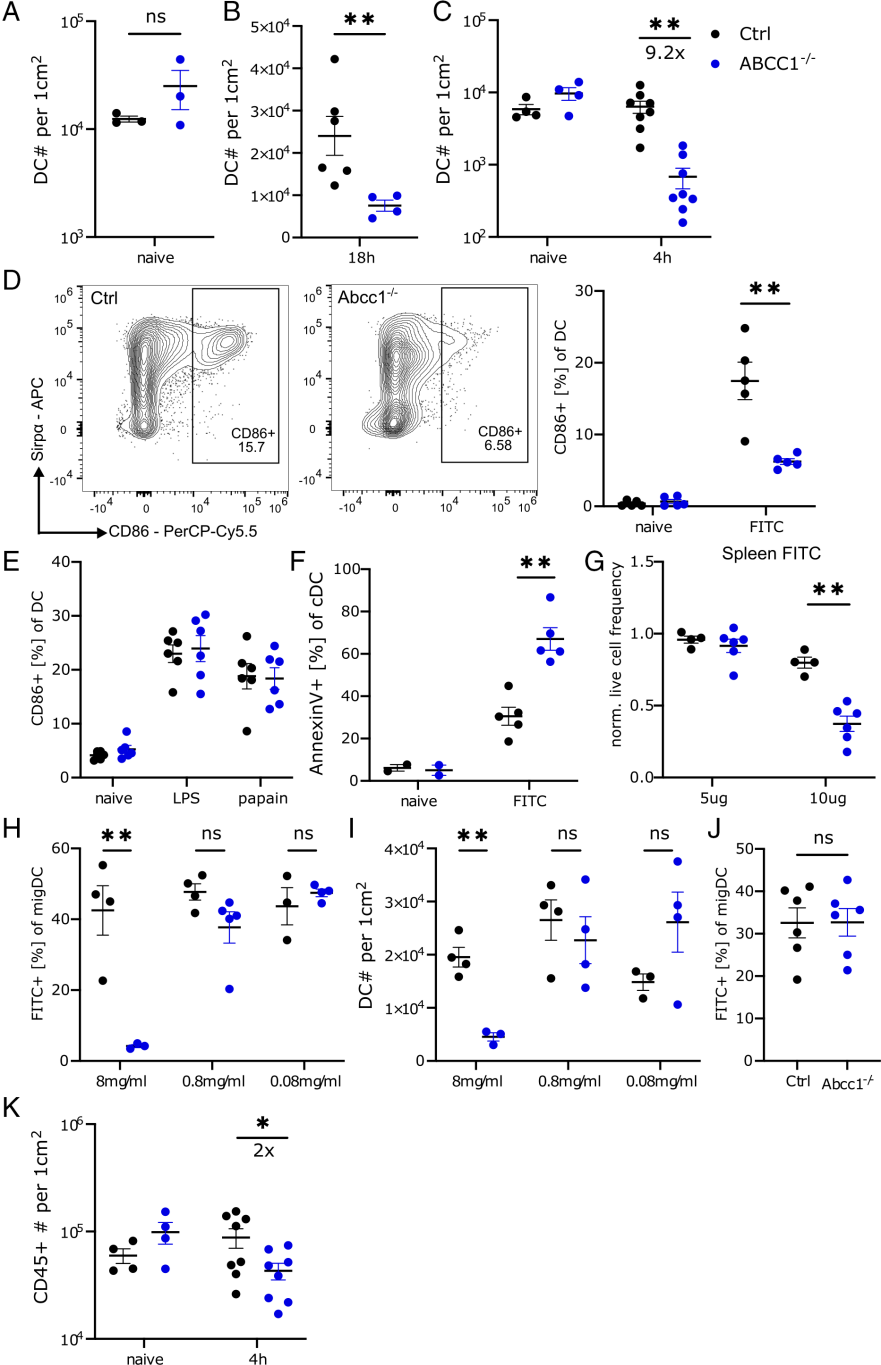
FITC treatment induces apoptosis in ABCC1-deficient DCs. (*A*) Quantification of skin DCs in control and ABCC1-deficient naive mice. (*B*) Quantification of skin DCs in control and ABCC1-deficient mice 18 h after FITC treatment. (*C*) Quantification of skin DCs in control and ABCC1-deficient mice 4 h after FITC treatment. (*D*) Representative flow cytometry plot and quantification of CD86 expression on skin DCs in control and ABCC1-deficient mice. Skin was analyzed 4 h after FITC treatment on the skin. (*E*) Quantification of CD86 expression on skin DCs in control and ABCC1-deficient mice 4 h after papain or LPS treatment. (*F*) Quantification of Annexin-V staining on skin DCs in control and ABCC1-deficient mice 4 h after FITC treatment. (*G*) Quantification of normalized live cell frequency from splenocytes of control and ABCC1-deficient mice 24 h after FITC treatment ex vivo. Live frequency is normalized to solvent control. (*H*) Quantification of sdLN FITC+ migratory DCs in control and ABCC1-deficient mice. sdLN were analyzed 18 h after skin treatment with the indicated FITC concentrations. (*I*) Quantification of skin DCs in control and ABCC1-deficient mice 18 h after FITC treatment as in *I*. (*J*) Quantification of sdLN FITC+ migratory DCs in control and ABCC1-deficient mice. sdLN were analyzed 3 d after 0.08 mg/mL FITC treatment on the skin. (*K*) Quantification of skin CD45+ cells in control and ABCC1-deficient mice 18 h after FITC treatment. All data are representative of at least two independent experiments (n ≧ 3 mice per experiment). Error bars indicate SEM, and statistical analysis was done with Student’s *t* test. ns = not significant; **P-value < 0.01.

We next tested if alterations in DC behavior following FITC treatment might manifest within the skin. ABCC1-deficient mice harbored normal numbers of skin DCs at homeostasis (Fig. 2*A*) but showed a markedly reduced recovery of skin DCs 18 h post-FITC application (Fig. 2*B*). The reduction in skin DC numbers in ABCC1-deficient mice was detectable as early as 4 h posttreatment, arguing against the reduction being secondary to increased egress of DCs from the tissue (Fig. 2*C*). Skin LCs were also reduced 4 h after FITC treatment (*SI Appendix*, Fig. S2 *A* and *B*). Moreover, activated DCs (CD86+) were almost entirely absent in FITC-treated ABCC1-deficient mouse skin (Fig. 2*D*). This observation could not be reconciled by a defect in the activation capacity, as ex vivo maturation (*SI Appendix*, Fig. S2*C*) and activation following exposure to lipo-polysaccharide (LPS) and papain occurred comparably for ABCC1-deficient and control cells (Fig. 2*E*).

We observed markedly greater accumulation of FITC in several cell types, following both in vivo and ex vivo FITC exposure, in ABCC1-deficient compared to control mice (*SI Appendix*, Fig. S2 *D* and *E*). Given the elevated intracellular accumulation of FITC and the concomitant loss of DCs from the skin, we speculated that the treatment might have induced apoptosis in the DCs. Indeed, we detected increased Annexin-V surface staining of ABCC1-deficient DCs following FITC treatment (Fig. 2*F*). To assess whether this phenomenon could be attributed to secondary effects, we cultured splenocytes from control and ABCC1-deficient mice in the presence of FITC. Consistent with our in vivo data, ABCC1-deficient splenocytes exhibited reduced viability compared to control cells following incubation with FITC (Fig. 2*G*). We next compared the dose sensitivity of the FITC-induced effects on skin DCs. 10-fold or 100-fold lower amounts of FITC were sufficient to promote control skin DC migration (Fig. 2*H*). At these lower FITC doses, ABCC1 deficiency did not impact DC migration and skin DC numbers were not reduced by the treatment (Fig. 2*I*). To exclude the possibility that FITC dilution merely delayed cell death, we measured the frequency of FITC+ DCs 3 d posttreatment with the reduced dose of FITC. No significant difference was observed in FITC+ DC frequency between ABCC1-deficient and control mice (Fig. 2*J*).

**Fig. 3.**
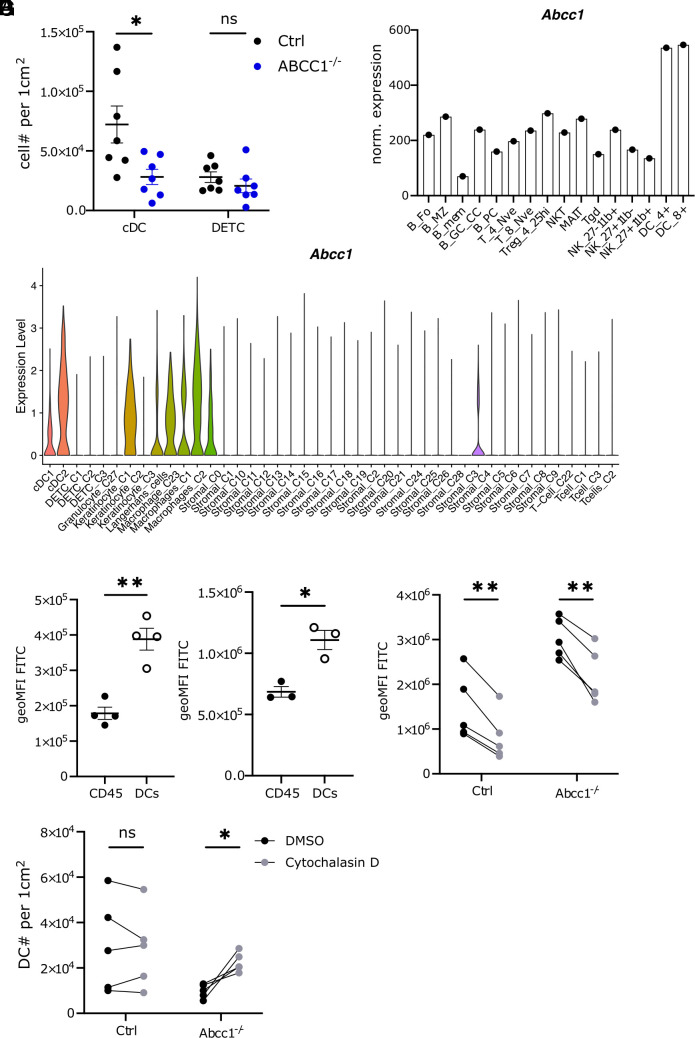
DCs have higher dependency on ABCC1 for survival. (*A*) Quantification of skin DETC and DCs in control and ABCC1-deficient mice 4 h after FITC treatment. (*B*) Normalized expression level of *Abcc1* in immune cells (immgen.org). (*C*) Normalized expression level of *Abcc1* in skin single-cell data displayed as a violin plot. Cells are grouped by cell type. (*D*) Quantification of FITC geoMFI of total CD45+ cells or DCs from spleen 24 h after FITC treatment ex vivo. (*E*) Quantification of FITC geoMFI of total CD45+ cells or DCs from skin 4 h post FITC treatment. (*F*) Quantification of FITC geoMFI of DCs from skin 4 h after FITC treatment. Groups were either treated with DMSO or Cytochalasin D. (*G*) Quantification of skin DCs in control and ABCC1-deficient mice 4 h after FITC treatment. Groups were either treated with DMSO or Cytochalasin D. All data are representative of at least two independent experiments (n ≧ 3 mice per experiment). Error bars indicate SEM, and statistical analysis was done with Student’s *t* test. ns = not significant; **P-value < 0.01; ***P-value < 0.001.

Importantly, the reduction in skin DC numbers was more pronounced than that observed for other cell types. While DCs were reduced 9.2-fold 4 h post-FITC treatment in ABCC1-deficient mice (Fig. 2*C*), the total CD45+ cell population decreased by only twofold (Fig. 2*K*). To further investigate differential sensitivity to FITC-induced cell death, we compared DCs with dendritic epidermal T cells (DETCs), cells that are expected to be highly exposed to FITC due to their epidermal localization. Treatment of mice with 2 mg/mL FITC revealed a significant reduction in DC numbers in ABCC1-deficient mice, whereas DETC numbers remained comparable to control mice (Fig. 3*A*). These results suggest that DCs are more susceptible than lymphocytes to FITC-induced apoptosis.

Analysis of publicly available spleen RNA-seq data [www.mmgen.org, (15)] indicated that DCs exhibited higher *Abcc1* expression than lymphocyte populations (Fig. 3*B*). Analysis of scRNA-seq data generated from mouse skin cells (16–18) revealed that skin DCs, LCs, and macrophages expressed *Abcc1* at higher levels than lymphoid cells or stromal cells (Fig. 3*C* and *SI Appendix*, Fig. S3 *A* and *B*). Collectively, these data suggest a greater requirement for ABCC1 function in DCs, LCs, and likely macrophages compared to other immune cells. In line with this notion, we observed that following FITC exposure in vivo and ex vivo, DCs harbored significantly higher FITC levels than the other immune cells that were analyzed (Fig. 3 *D* and *E*).

Considering the differences in myeloid and lymphoid cell biology and the elevated intracellular FITC levels, we speculated that high endocytic activity (micropinocytosis), a hallmark of DCs, might underlie our observations. To investigate this possibility, we pharmacologically blocked micropinocytosis with the actin polymerization inhibitor Cytochalasin D (19, 20). This treatment reduced FITC levels in both control and ABCC1-deficient DCs in the skin (Fig. 3*F*), but not significantly in DETCs (*SI Appendix*, Fig. S3*C*), indicating micropinocytosis may be responsible for the higher FITC levels in DCs. Furthermore, inhibiting micropinocytosis partially rescued DCs from cell death, as evidenced by increased DC numbers in the skin of FITC-painted ABCC1-deficient mice (Fig. 3*G*). We do not exclude the possibility that Cytochalasin D treatment impacts additional cellular processes that influence DC survival.

**Fig. 4.**
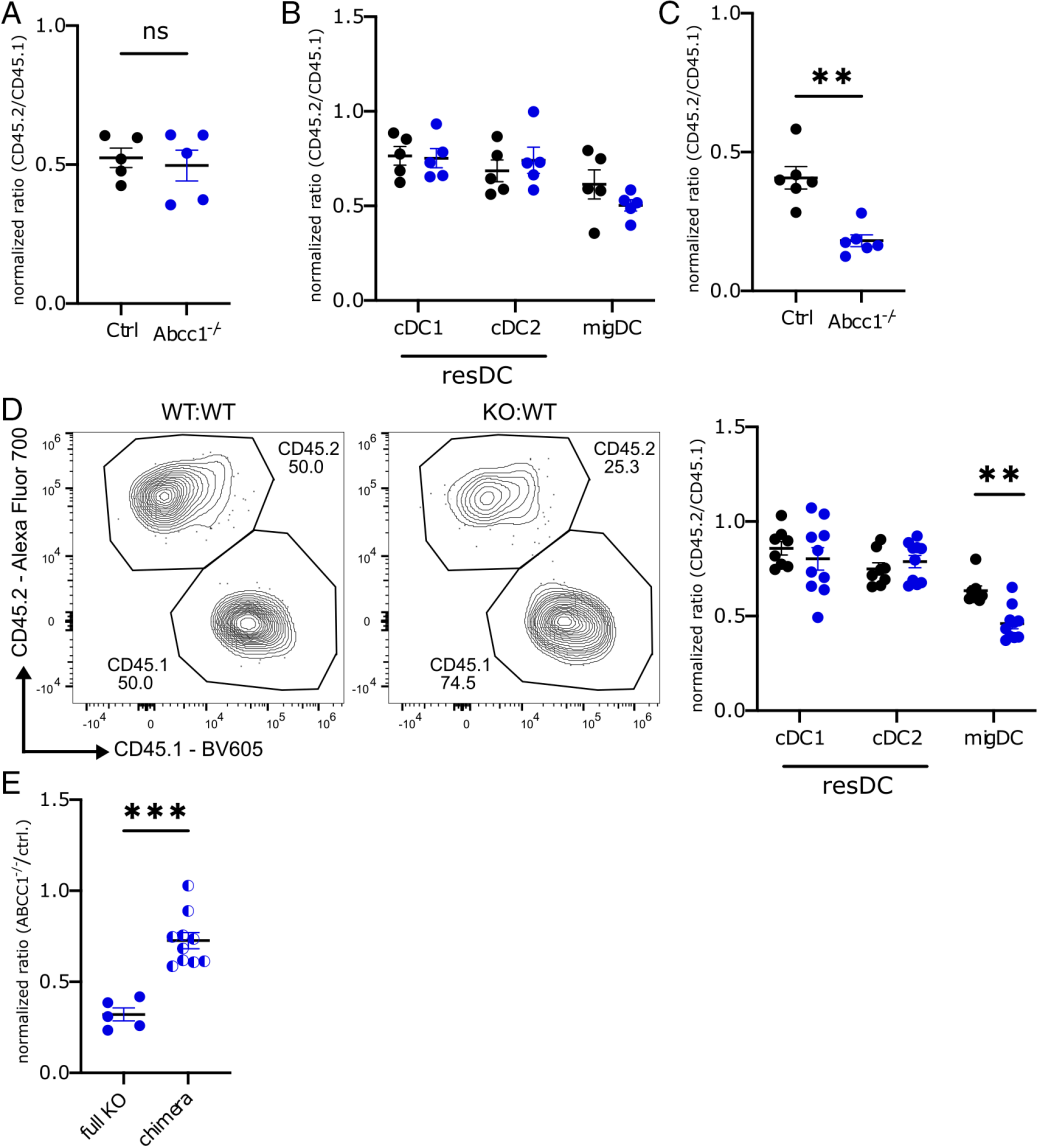
ABCC1 acts cell intrinsically and cell extrinsically to promote DC survival. (*A* and *B*) Quantification of normalized CD45.2/CD45.1 ratio of skin DCs (*A*) or sdLN DCs (*B*) in mixed BM chimeric mice with CD45.2 either representing control (Ctrl) or Abcc1^−/−^ cells and CD45.1 WT cells. (*C* and *D*) Quantification of normalized CD45.2/CD45.1 ratio as in *A* of skin DCs (*C*) and representative flow cytometry plot and quantification of normalized ratio of sdLN DCs (*D*) 18 h after FITC treatment. (*E*) Normalized ratio of Abcc1^−/−^ to control sdLN DCs in FITC painted nonchimeric (full KO) and mixed chimeric mice. Data in *E* are from Figs. 1*C* and 4*D*. For nonchimeric mice, the ratio is between different mice (Abcc1^−/−^ vs. WT), whereas for mixed chimeric mice, the ratio is for Abcc1^−/−^ vs. WT cells in the same chimera. (*A*–*D*) CD45.2/CD45.1 DC ratio is normalized to CD19+ cell ratio from the sdLN. All data are representative of at least two independent experiments (n ≧ 3 mice per experiment). Error bars indicate SEM, and statistical analysis was done with Student’s *t* test. *P-value < 0.05; **P-value < 0.01.

Our data indicate that ABCC1 protects cells from apoptosis with the in vitro data providing evidence for a cell-intrinsic mechanism. To further test the cell-intrinsic role of the transporter, we generated mixed chimeric mice by reconstituting lethally irradiated hosts with a 1:2 mixture of control or ABCC1-deficient BM cells and congenically marked wild-type BM cells. In the absence of challenge, the proportion of control or ABCC1-deficient skin DCs in the chimeras was comparable in skin (Fig. 4*A*) and sdLN (Fig. 4*B*). Following the FITC challenge, there was a reduction in the proportion of ABCC1-deficient DCs in skin (Fig. 4*C*) and of ABCC1-deficient migratory DCs in sdLN (Fig. 4*D*). These data indicate that ABCC1 acts in a DC-intrinsic manner. However, the reduction in DCs was less prominent in the mixed chimera setting than in fully ABCC1-deficient mice (Fig. 4*E*), suggesting that ABCC1 also promotes DC survival in a cell-extrinsic manner.

**Fig. 5.**
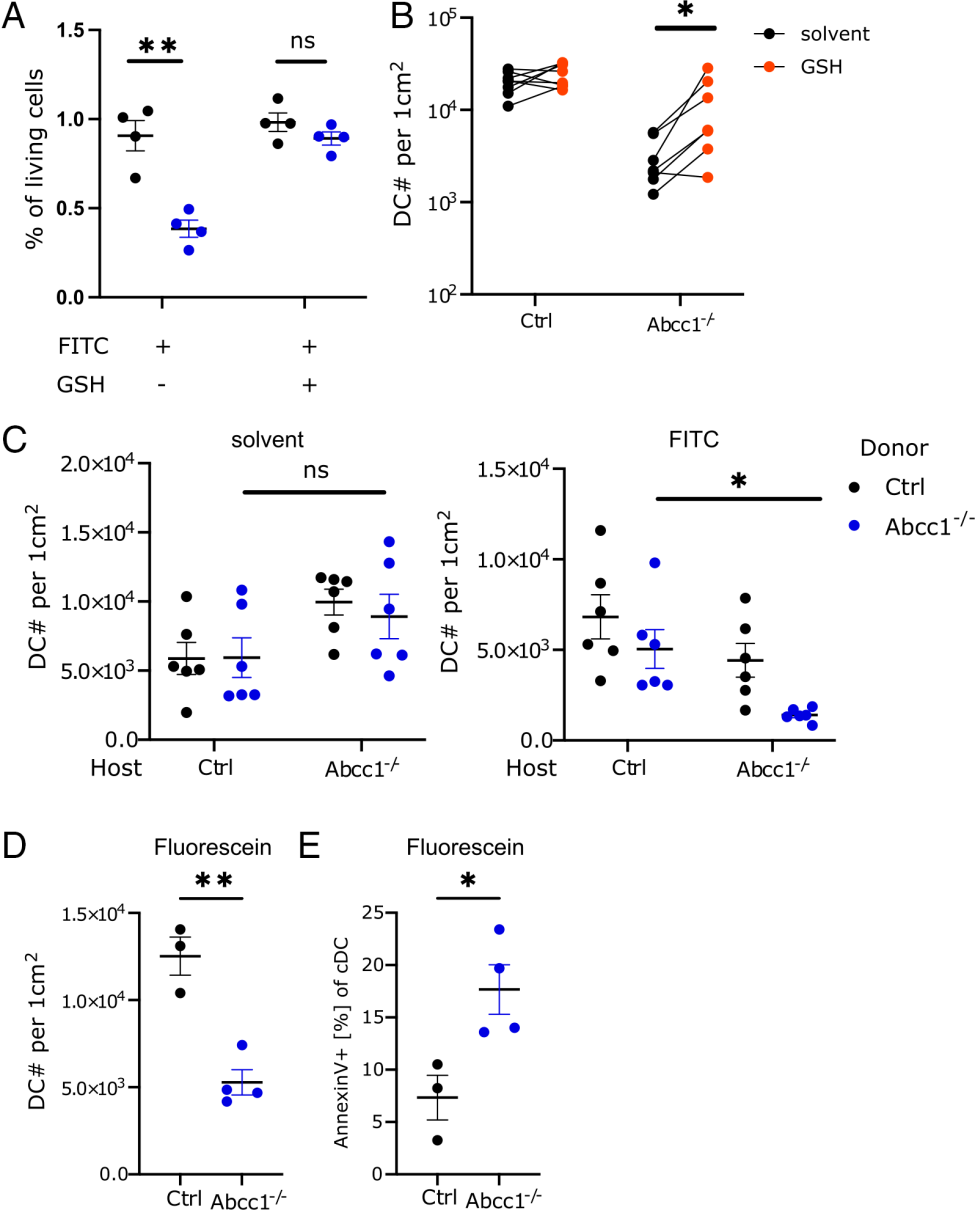
Activity of extracellular GSH in protecting DCs from FITC and sensitivity of ABCC1-deficient DCs to fluorescein. (*A*) Quantification of normalized live cell frequency from splenocytes of control and ABCC1-deficient mice 18 h after FITC (10 μg/mL) and GSH (50 μg/mL) treatment ex vivo. (*B*) Quantification of skin DCs in control and ABCC1-deficient mice 4 h after FITC and either saline or 1 mg GSH treatment. (*C*) Quantification of skin DCs with host mice either representing control or Abcc1^−/−^ and DCs being derived from control or Abcc1^−/−^ donor BM. The left plot depicts solvent treated mice and right depicts mice 4 h after FITC treatment. (*D*) Quantification of skin DCs in control and ABCC1-deficient mice 4 h after fluorescein treatment. (*E*) Quantification of Annexin-V staining on skin DCs in control and ABCC1-deficient mice 4 h after fluorescein treatment. All data are representative of at least two independent experiments (n ≧ 3 mice per experiment). Error bars indicate SEM, and statistical analysis was done with Student’s *t* test. ns = not significant.

One function of ABCC1 is the transport of GSH across the plasma membrane (2). Extracellular GSH contributes to the antioxidant properties of skin (21). We speculated that extracellular GSH might play a role in protecting DCs from FITC-induced apoptosis by interacting with the ITC group of FITC and limiting its entry into the cells. To this end, we cultured splenocytes together with FITC and added GSH to the culture. Adding GSH reduced the FITC-induced apoptosis (Fig. 5*A*). To test whether extracellular GSH has a protective effect in vivo, we subcutaneously treated control and ABCC1-deficient mice with GSH immediately before FITC painting. As before, we could recover less DCs from the skin in ABCC1-deficient mice 4 h post FITC painting compared to control mice (Fig. 5*B*). Treating with GSH ameliorated the phenotype and partially rescued the skin DCs from apoptosis (Fig. 5*B*). To further test the activity of ABCC1 in protecting DC viability cell-extrinsically, we reconstituted ABCC1-deficient and control mice with a mixture of ABCC1-deficient and control BM-cells. This set-up allowed us to investigate the survival of ABCC1-deficient cells, comparing a skin environment almost completely lacking GSH exported by ABCC1 to physiological extracellular GSH levels. FITC painting revealed that the ABCC1-deficient DC survival in the skin was reduced in a GSH-deprived environment compared to control mice with an intact ABCC1 GSH export capacity (Fig. 5*C* and *SI Appendix*, Fig. S4*A*). Together, these data provide evidence for the importance of extracellular GSH exported by ABCC1 in protecting DCs from FITC-induced cell death.

Finally, we tested the selectivity of ABCC1 in mediating DC protection from FITC-induced toxicity. ABCC1 deficiency did not affect DC sensitivity to benzylisothiocyanate (BITC) (*SI Appendix*, Fig. S4*B*) or several other ITCs (PEITC, AITC, unpubl. obs.). To investigate whether FITC’s toxicity in ABCC1-deficient cells was independent of its ITC group, we treated mice with fluorescein. Fluorescein had a similar propensity as FITC to induce skin DC cell loss (Fig. 5*D*) and Annexin-V staining showed that this occurred by apoptosis (Fig. 5*E*). DETC were minimally altered in fluorescein sensitivity by the loss of ABCC1 (*SI Appendix*, Fig. S4*C*). These data suggest that ABCC1 protects DCs from FITC toxicity at least in part by preventing toxic intracellular accumulation of fluorescein, independent of FITC’s ITC group. We then tested skin DC sensitivity to two chemotherapeutic agents that can be substrates for ABCC1, doxorubicin and vincristine (2). We did not observe increased sensitivity of ABCC1-deficient DCs to cell death induced by these chemicals. (*SI Appendix*, Fig. S4 *D* and *E*). These data indicate ABCC1 has a role in protecting skin DCs from a select class of reactive chemicals.

## Discussion

Together our findings highlight the importance of ABCC1, also known as multidrug resistance transporter protein-1 (MRP1), in enabling skin DCs to resist the toxic effects of FITC and fluorescein. Furthermore, we provide evidence for the extracellular function of GSH in reducing FITC reactivity and thereby promoting skin DC viability.

In earlier work that identified a role for ABCC1 in the migratory response of skin DCs following FITC exposure, a model was proposed involving ABCC1-exported LTC4 enabling CCR7-dependent migration to CCL19 (9). However, the CCL19 findings were based on ex vivo data and DC migratory responses are challenging to study ex vivo due to spontaneous DC maturation (22). Analysis of CCL19-deficient mice does not support a requirement for CCL19 during FITC-induced skin DC migration in vivo [(14) and this report]. Moreover, we found that skin exposure to low amounts of FITC induced equal migration of ABCC1-deficient and control DCs. A study tracing lung DC migration to the dLN following fluorescent bead exposure also showed no difference comparing ABCC1-deficient and control mice (23). These findings favor the general conclusion that ABCC1 is not directly required for CCR7-mediated DC migration from tissue to dLNs.

Immature DCs undergo micropinocytosis and are estimated to take up 1,000 μm^3^ or 40% of their volume every hour (24). By internalizing more interstitial fluid than other cell types DCs may experience increased entry of environmental chemicals to the cytosol where they could react with essential intracellular proteins, thereby making DCs more sensitive to chemical-induced toxicity. The high sensitivity of ABCC1-deficient DCs to FITC and fluorescein may also reflect their strong metabolic needs as they undergo maturation and migration within hours of exposure to these agents. Highly metabolically active cells may be more dependent on the intactness of their intracellular proteins and thus may experience stresses more readily than cells that are in a quiescent state.

While FITC is known to react with and label proteins in the plasma membrane, it can also enter cells (25). In this regard, it is notable that FITC has a MW below 500 Da and thus within a size range that permits passage across membranes (26, 27). This is in accord with our finding that deficiency in ABCC1, a transporter that effluxes molecules out of the cytosol, causes cells to accumulate higher amounts of FITC. An open question remaining is how FITC or fluorescein are cytotoxic. Future studies will need to address the molecular mechanism and whether a DC-specific biological pathway is disrupted by these chemicals.

Glutathione (GSH), the most abundant thiol-containing molecule in cells, reacts with ITCs and modulates their toxicity (6). GSH is also abundant extracellularly in skin (21). ABCC1 contributes to extracellular GSH (2) and by reacting with FITC this may limit its access to cells. Our mixed chimera and in vitro culture data establish a cell-intrinsic role for ABCC1 in limiting FITC accumulation in DCs and other cells, in accord with a role in efflux of FITC that reached the cytosol and likely reacted with GSH. However, our data also indicate a role for extracellular GSH in restraining FITC entry into cells, thus providing evidence that ABCC1 helps establish a chemical buffer zone in the skin. While GSH is the most likely ABCC1 substrate protecting cells from FITC, ABCC1 can transport a variety of other substrates, and we do not exclude the possibility that it influences the extracellular milieu in additional ways that protect cells from reactive chemicals.

By removing an important exporter of diverse cargo, we show that DCs have greater sensitivity than other cell types tested to one type of environmental chemical. The lack of ABCC1-deficient DC sensitivity to several other chemicals we tested may reflect other members of the ABC transporter family compensating for the loss of ABCC1. Future experiments will need to address the contribution of multiple ABC transporter genes to DC physiology and resistance to environmentally induced toxicity. Humans are exposed to a huge diversity of environmental chemicals including many new synthetic molecules. It may prove valuable for advancing understanding of the potential impact of these molecules on the immune system to screen them broadly for negative effects on control and ABC transporter-deficient DC viability.

## Materials and Methods

### Animals.

Breeding was done in an internal colony, housed under 12-h light:12-h dark cycles and given ad libitum access to food and water. Then, 7 to 18-wk-old mice of both sexes were used for experiments. Abcc1^−/−^ mice and Ccl19^−/−^ mice were generated as described previously and were on a C57BL6/J (B6) background (28, 29). B6 mice and CD45.1 congenic B6 mice (B6.SJL-PtprcaPepcb/BoyCrCrl) were bred internally. Littermate and cage mate controls were used for experiments. Animals were housed in a pathogen-free environment in the Laboratory Animal Resource Center at the University of California, San Francisco, and all experiments conformed to ethical principles and guidelines that were approved by the Institutional Animal Care and Use Committee.

### Treatment of Mice.

Mice were shaved with a clipper at the dorsal back skin before treatment. GSH (5 mg), Cytochalasin D (1 μg), papain (50 μg), and LPS (5 μg) were injected subcutaneously in 50 μL at the stated concentrations dissolved in saline or saline with 1% DMSO. FITC, fluorescein, BITC, vincristine, and doxorubicin were applied in 25 μL to the shaved dorsal skin at stated concentrations. For topical applications, the chemicals were dissolved in a 1:1 mix of Dibutylpthalate:Acetone and freshly prepared before treatment.

### BM-Chimera Generation.

Mice, where indicated, were lethally irradiated with 900 rads gamma-irradiation (split dose separated by 4 h) and then i.v. injected with 5 × 10^6^ BM cells. To generate mixed BM-chimera, BM cells from each donor mouse were counted using a hemocytometer and 5 × 10^6^ BM-cells were transferred in total with the mixing ratio indicated in the main text. Mice were analyzed 6 to 8 wk postreconstitution.

### Ear-Crawl Out Assay.

Ears were harvested from mice and split to separate ventral and dorsal half. Each half was placed, dermis facing down, onto 1 mL of RPMI with 1 mM Hepes, and 10% fetal bovine serum, supplemented with P/S, GlutMax, and NEAA in a 6-well plate. The tissue was incubated for 24 h, and medium was collected. Dendritic cell numbers in media were counted using Cytek aurora after staining with flow antibodies.

### Cell Isolation LN.

Mice were euthanized, LNs were isolated and placed in 1 mL digestion buffer (composed of 10 μg/mL collagenase IV and 0.1 mg/mL DNase in RPMI with 1 mM Hepes, and 2% fetal bovine serum). The LNs were rotated at 37 °C for 60 min. Afterward, a single-cell suspension was generated using a pestle and the cells were transferred into a staining buffer (PBS containing 2% NBCS and 0.05% Sodium Azide) for cell counting and staining.

### Cell Isolation Skin.

A shaved skin patch of 1 cm × 1 cm was dissected, minced finely with scissors, resuspended in 1 mL of digestion media (composed of 0.25 mg/mL Liberase TM, 0.5 mg/mL hyaluronidase, and 0.1 mg/mL DNase in RPMI with 1 mM Hepes, and 2% fetal bovine serum), and incubated in a heated shaker at 37 °C at 1,000 rpm for 60 min. An additional 1 mL of RPMI/Hepes/FBS media containing 1 mM EDTA was added, and the suspension was vortexed and then strained into a 50 mL conical tube through a 70 μm strainer. Another 10 mL of medium was added to thoroughly wash the cell strainer and the suspension was centrifuged, resuspended in a staining buffer (PBS containing 2% NBCS and 0.05% Sodium Azide) for cell counting and staining. Cell counts per 1 cm^2^ of digested skin were derived from Cytek aurora analyzer and calculated based on acquired volume.

### Splenocyte Isolation and Culture.

Spleens were harvested, minced finely with scissors, resuspended in 1 mL of digestion media (composed of 10 μg/mL collagenase IV and 0.1 mg/mL DNase in RPMI with 1 mM Hepes, and 2% fetal bovine serum). The spleens were rotated at 37 °C for 60 min. Afterward, a single-cell suspension was generated using a pestle. Red blood cells were lysed using RBC lysis buffer and afterward the single-cell suspension was transferred into a staining buffer (PBS containing 2% NBCS and 0.05% Sodium Azide). Where indicated, splenocytes were cultured in culture media (composed of RPMI with 1 mM Hepes, and 10% fetal bovine serum, supplemented with P/S, GlutMax, and NEAA).

### Flow Cytometry Analysis.

Single-cell suspension obtained from tissues were used for flow cytometry analysis using Cytek aurora. The data were analyzed and visualized using Flowjo software (v10). Cells were surface stained with Zombi NIR stain (Biolegend), anti-γδTCR (Biolegend; clone: GL3; BV650), anti-CD11b (BD; clone: M1/70; BUV493), anti-CD11c (Biolegend; clone: N418; PE/ Spark NIR 685/ APC), anti-CD19 (BD; clone: 1D3; BUV563), anti-IA/IE (BD; clone: M5/114; BUV805/ PerCP-cy5.5), anti-Epcam (Biolegend; clone: G8.8; BV711), anti-CD64 (Biolegend; clone: W18349C; PE-Cy7), anti-CD86 (Biolegend; clone GL-1; PerCP-Cy5.5), anti-Sirpa (Biolegend; clone: P84; PE-Dazzle594/ APC), anti-XCR1 (Biolegend; clone: ZET; AL647), anti-CD45.2 (Biolegend; clone: 104; BUV395/ AL700), anti-CD45.1 (Biolegend; clone: A20; AL700/ BV605), Ly6G (Biolegend; clone: 1A8; APC-Cy7), CD4 (BD; clone: GK1.5; BUV615), CD8a (BD; clone: 53-6.7; BUV737).

### CCR7 Stain.

Anti-CCR7 antibody (Biolegend; clone: 4B12; biotinylated) was incubated with cells for 1 h at 37 °C. Afterward, cells were washed with PBS containing 2% NBCS and 0.05% Sodium Azide two times. SA conjugated to PE (Biolegend) was incubated together with additional surface antibodies in a staining buffer for 30 min on ice.

### Annexin-V Staining.

After surface antibody staining, cells were washed with Annexin-V staining buffer (BD) and stained in Annexin-V staining buffer with PE-conjugated Annexin-V (BD) for 15 min at RT. Cells were washed with Annexin-V staining buffer and analyzed using Cytek aurora.

### RNA-Sequencing Data Processing and Analysis.

For the presented bulk and scRNA-sequencing results, published datasets were analyzed running a customized R script with Seurat v.4.2 for single-cell sequencing. Raw counts of the published data were used [GEO Accession: GSE266028 and GSE266027 (16–18)] or normalized expression values were retrieved from immgen.org (15).

### Statistical Analysis.

Apart from the genomic data, all biological data were analyzed using Prism 10 software (GraphPad) by two-tailed paired Student’s *t* test or two-tailed unpaired Student’s *t* test.

## Supplementary Material

Appendix 01 (PDF)

## Data Availability

All study data are included in the article and/or *SI Appendix*. Previously published data were used for this work [immgen.org (15); GSE266028, GSE266027 (16–18)].
